# Initial Learning Curve for Robot-Assisted Total Knee Arthroplasty in a Dedicated Orthopedics Center

**DOI:** 10.3390/jcm12216950

**Published:** 2023-11-06

**Authors:** Serban Dragosloveanu, Mihnea-Alexandru Petre, Bogdan Sorin Capitanu, Christiana Diana Maria Dragosloveanu, Romica Cergan, Cristian Scheau

**Affiliations:** 1 The “Carol Davila” University of Medicine and Pharmacy, 050474 Bucharest, Romania; 2 Department of Orthopaedics, “Foisor” Clinical Hospital of Orthopaedics, Traumatology and Osteoarticular TB, 021382 Bucharest, Romania; 3 Department of Ophthalmology, Clinical Hospital for Ophthalmological Emergencies, 010464 Bucharest, Romania; 4 Department of Radiology and Medical Imaging, “Foisor” Clinical Hospital of Orthopaedics, Traumatology and Osteoarticular TB, 021382 Bucharest, Romania

**Keywords:** robotic-assisted surgery, total knee arthroplasty, learning curve, ROSA

## Abstract

*Background and objectives:* Our study aimed to assess the learning curve for robot-assisted (RA) total knee arthroplasty (TKA) in our hospital, compare operative times between RA-TKAs and manual TKAs, and assess the early complications rate between the two approaches. *Methods:* We included 39 patients who underwent RA-TKA and 45 control patients subjected to manual TKA in the same period and operated on by the same surgical staff. We collected demographic and patient-related data to assess potential differences between the two groups. *Results:* No statistical differences were recorded in regard to age, BMI, sex, Kellgren–Lawrence classification, or limb alignment between patients undergoing RA-TKA and manual TKA, respectively. Three surgeons transitioned from the learning to the proficiency phase in our study after a number of 6, 4, and 3 cases, respectively. The overall operative time for the learning phase was 111.54 ± 20.45 min, significantly longer compared to the average of 86.43 ± 19.09 min in the proficiency phase (*p* = 0.0154) and 80.56 ± 17.03 min for manual TKAs (*p* < 0.0001). No statistically significant difference was recorded between the global operative time for the proficiency phase TKAs versus the controls. No major complications were recorded in either RA-TKA or manual TKA groups. *Conclusions:* Our results suggest that experienced surgeons may adopt RA-TKA using this platform and quickly adapt without significant complications.

## 1. Introduction

Total knee arthroplasty (TKA) is a highly effective orthopedic treatment for patients with symptomatic end-stage knee osteoarthritis (OA), a common condition in the elderly population and one of the leading causes of disability [1,2,3]. Recent studies have shown that implant survivorship may be higher than 90% at 10 years of follow-up [4,5], with a patient satisfaction rate within the range of 75% to 92% [6,7,8,9]. Despite advances in materials used for implants, improvement in designs, surgical approaches, recovery programs, thromboembolic and antibiotic prophylaxis patient satisfaction rate is lower compared to patients following total hip arthroplasty [10,11,12]. A variety of surgeon-dependent variables may affect patient outcomes, such as implant positioning, flexion-extension gaps, soft tissue preservation, etc. [13,14].

Robotic technology has emerged as a technological method aiming to eliminate human error and improve patient outcomes. The first robotic-assisted TKA (RA-TKA) was performed in 1988 in the United Kingdom using the ACROBOT^®^ robotic system [15]. The main advantages of robotic surgery over manual TKA are the reduction of postoperative alignment outliers, improvement in gap balance, and increased reproducibility of postoperative knee biomechanics [16,17].

Robots used for TKA are either semi-active or fully active [16]. Semi-active robotic systems enable the surgeon to maintain control over bone resection and implant positioning and provide live intraoperative assistance; examples include ROSA^®^ (Zimmer-Biomet, Warsaw, IN, USA), Mako (Stryker, Kalamazoo, MI, USA), and NAVIO (Smith & Nephew, London, UK). Fully active robotic systems work autonomously to perform femoral and tibial bone resections (e.g., DigiMatch™ ROBODOC^®^ surgical system, Curexo Technology Corp, Fremont, CA, USA), while the surgeon supervises the bone resection and may stop the process if needed [18,19].

The Robotic Surgical Assistant (ROSA^®^) Knee robot was developed by Zimmer-Biomet in collaboration with MedTech [20]. This system is an interactive robotic platform with a robotic arm that allows the cutting guides to be optimally positioned based on the intraoperative plan determined using navigation jigs. The surgeon performs the surgical approach, retractors positioning, and sawing process [21].

There are, however, several reported controversies about RA-TKA, including the high cost of installation, compatibility with certain prosthetic designs, and potentially higher radiation exposure [22]. Moreover, availability for the entirety of surgical staff and accommodation to robotic or navigation systems were also reported as limitations of these systems [23].

The primary objective of this study was to assess the learning curve of experienced high-volume orthopedic surgeons performing RA-TKA using the ROSA^®^ platform in our dedicated orthopedics hospital. Additionally, we compared the operative times and rate of complications between RA-TKA and manual TKA surgeries. We hypothesized that the cumulative experience of the surgical staff in performing RA-TKA would lead to improved operative times with comparable surgical complications.

## 2. Materials and Methods

### 2.1. Study Population

Our retrospective cohort study included 84 patients who underwent TKA in our hospital between March and September 2023. The study group consisted of a subset of 39 consecutive patients undergoing RA-TKA and 45 consecutive patients undergoing manual TKA. All patients subjected to RA-TKA during the specified period were considered for this study, as well as all patients where manual TKA was performed in the same time intervals by the same surgical staff training with ROSA^®^ as controls for comparing the operative time. Patients for each surgeon were randomly assigned either to the RA-TKA or the manual TKA group and scheduled for surgery based on the availability of the ROSA^®^ system (Tx2 version 1.2). The inclusion criteria were patients over 18 years old with symptomatic end-stage OA and correctable varus/valgus deformity of <15 degrees. The exclusion criteria were patients with tumoral involvement of the knee, inflammatory arthritis, neurological conditions affecting knee mobility, knee instability, or previous primary TKA.

We collected a series of demographic parameters such as age, sex, and BMI to ensure consistency between subgroups. Radiological scores and parameters such as the Kellgren–Lawrence score and the hip–knee–ankle angle were also employed.

Ethical approval was obtained from the Institutional Ethics Council. All procedures related to patient care and processing of their medical data were performed in accordance with the Declaration of Helsinki adopted in 1964 and its later amendments.

### 2.2. Surgical Protocol

**Figure 1:**
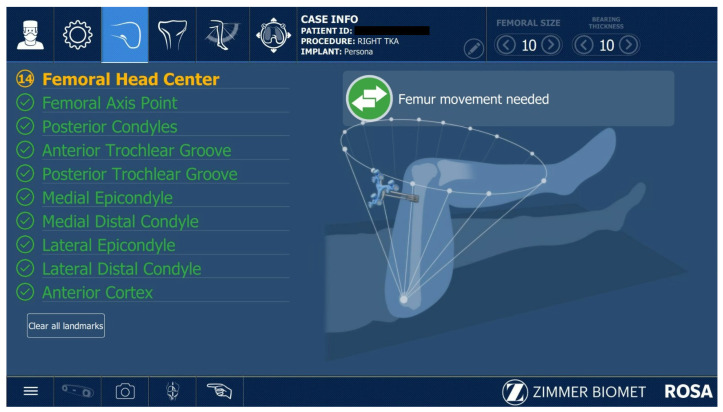
Assessing femoral head center.

**Figure 2:**
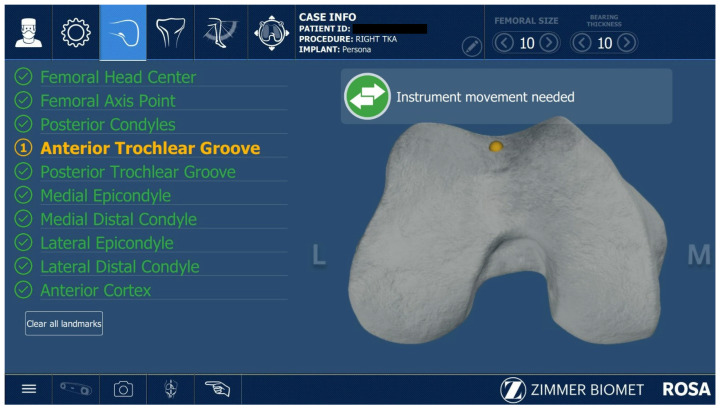
Capturing femoral bony landmarks.

**Figure 3:**
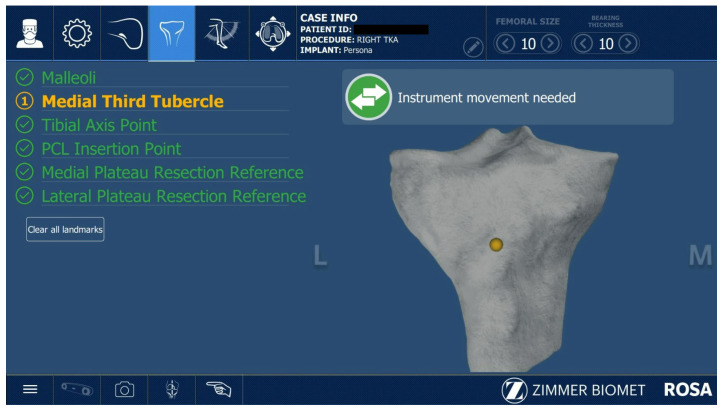
Capturing tibial bony landmarks.

All ROSA^®^-assisted TKAs were performed as described in the Zimmer Biomet ROSA surgical technique guide. After the initial correct positioning of the Robotic Unit and ROSA Optical Unit, the knee incision was performed with the preferred approach of the surgeon; in this case, subvastus and medial parapatellar approaches were used. The robotic arm preparation, case upload, robot settings, and draping were performed while the surgeon performed the surgical approach to the knee. The second step was installing the femoral and tibial trackers according to the ROSA^®^ surgical guide. Two femoral pins were installed in the proximal medial side of the distal femur. The two tibial pins were placed 4 fingers distally to the distal part of the wound, on the anteromedial tibial crest. The pins were 3.2 mm diameter ROSA^®^-specific pins. The next step was the assessment of the femoral head center of rotation (Figure 1) and digitalization of the bony landmarks (Figure 2 and Figure 3).

**Figure 4:**
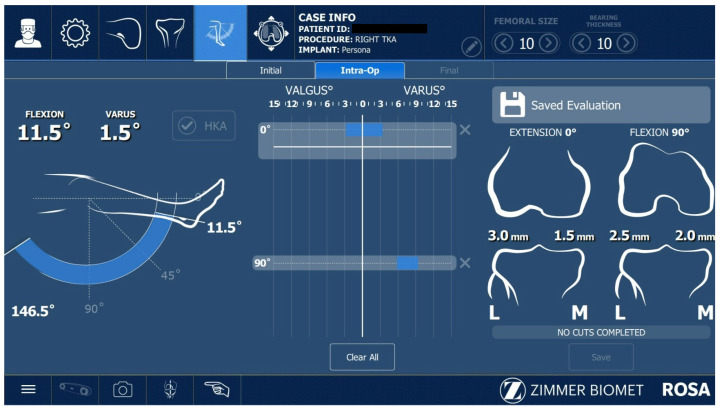
Assessing the range of motion of the knee, performing varus–valgus stress for ligament laxity.

After acquiring the femoral and tibial bony landmarks, the surgeon assessed the knee range of motion and performed varus and valgus laxity tests (Figure 4), while also removing large osteophytes that could have negatively influenced these tests.

**Figure 5:**
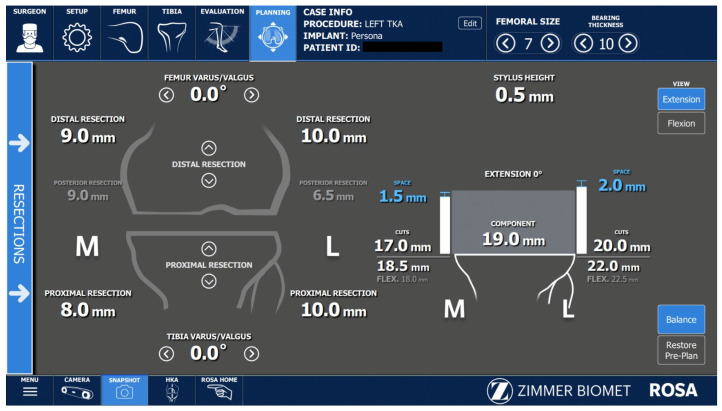
Balancing software (version 1.2).

All ROSA^®^-assisted TKAs performed in this study were imageless cases. The last step before performing the bone cuts was the ROSA^®^ software planning panel. After the appropriate balancing of the knee was achieved and the proper knee implant was selected, the surgeon performed the knee extension and flexion gaps (Figure 5). A restricted kinematic approach for balancing the knee was used in all cases.

The manual or “classic” TKAs were also performed taking into consideration the surgeons’ preferred surgical approach, i.e., the subvastus or medial parapatellar approach. For the femoral cut, all surgeons used the intramedullary guide, while for the tibial cut, both intra- and extramedullary NexGen^®^ guides were used, depending on the surgeon’s preference. For balancing the knee, each surgeon used their preferred TKA alignment approach between anatomic, mechanical, and restricted kinematics. After balancing the extension gap using the necessary soft tissue release technique, the next step was achieving a balanced flexion gap using the 4-in-1 cutting guide.

For all RA-TKAs, the Persona^®^ (The Personalized Knee^®^) PS prosthesis was used, while for all manual TKAs, the NexGen^®^ Complete Knee Solution PS was the preferred choice. The order of bone cuts was identical for RA-TKAs and manual TKAs. Tourniquet usage in TKA procedures remains a subject of ongoing debate in the orthopedic literature, with advocates for both sides [24,25,26]. In this regard, in our service and for the purposes of this study, the tourniquet was prepared but not inflated.

The surgical staff had prior training workshops for robotic-assisted TKAs, especially for the ROSA^®^ Knee robot. They were instructed on how to properly set up the surgical theater and ROSA^®^ system. The surgical staff had the same composition throughout this study. A total of 12 surgeons underwent training with ROSA^®^ and, for each of them, the operative time was assessed for every surgery. For each surgeon, both RA-TKAs and manual TKAs were performed using the same approach.

### 2.3. Complications

All patients were closely followed perioperatively by the case anesthesiologist and all complications, both related to the ROSA surgical technique and non-robotic-related situations, were recorded in the surgical protocol for each procedure.

### 2.4. Statistical Analysis

A cumulative summation (CUMSUM) analysis was performed to describe the learning curve and determine the inflection point, i.e., the transition from the learning phase into the proficiency (or experience) phase. The CUMSUM values for each surgeon were calculated as the running total of differences between the particular data point (i.e., surgical time) and the standardized target, which was established as the mean operative time for RA-TKAs performed by that surgeon. The *t*-test was used to compare continuous variables such as age or BMI across patient subgroups. Cohen’s d method was used to measure the size of the differences between the compared groups. The Chi-squared test was employed to determine whether the frequency of one parameter, such as gender or laterality, was significantly higher in a specific group. Analysis of variance was used to compare operative times across categories. The data were tested for normal distribution using the Shapiro–Wilk test. The statistical software used in this study was MedCalc^®^ Version 14.8.1 (MedCalc Software Ltd., Ostend, Belgium). Results were considered statistically significant for *p*-values < 0.05.

## 3. Results

A comparison of baseline demographics was performed and the results are depicted in Table 1 and Table S1. No statistical differences were recorded between the two subgroups. However, in terms of the mechanical axis of the lower limb, we identified six patients with valgus alignment in the manual TKA group, while no patients with valgus underwent RA-TKA in our study.

**Figure 6:**
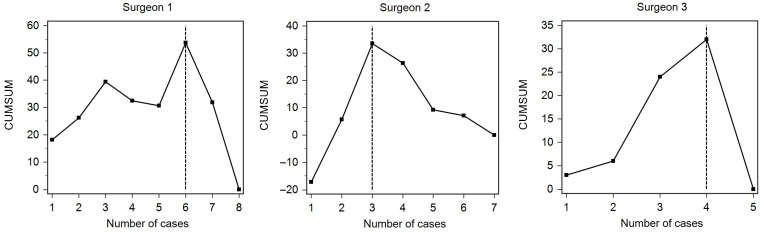
CUMSUM analysis of the learning curves for the three surgeons that passed from the learning phase to the proficiency phase. The transition between phases is marked by the vertical dashed line (i.e., inflection point).

The analysis of the operative times for RA-TKA showed that only three surgeons achieved the inflection point by the end of our study. The respective CUMSUM analysis for each surgeon is presented in Figure 6. The surgeons transitioned from the learning phase to the proficiency phase after 6, 3, and 4 cases, respectively.

A further comparative analysis was performed on the cases undergoing RA-TKA in the learning phase, cases performed in the proficiency phase, and patients operated by manual TKA in the same time period by the three surgeons (Table 2 and Table S2).

The overall operative time for the learning phase was significantly longer than both the times of surgeries performed in the proficiency phase and that of manual TKAs. No statistically significant difference was recorded between the global operative time for the proficiency phase TKAs versus the controls.

**Figure 7:**
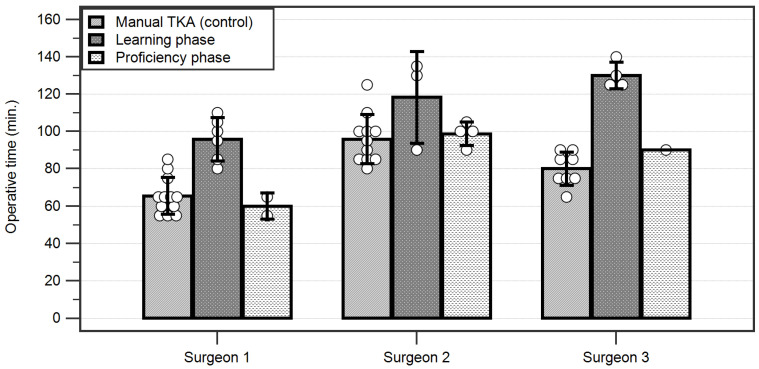
Operative times for each surgeon depicting their average times in the learning curve for robotic-assisted versus manual total knee arthroplasties. Each individual case is depicted by a circle. TKA = Total knee arthroplasty.

A detailed analysis of the operative times of each surgeon revealed that surgeries performed in the learning phase took significantly longer than those performed in the proficiency phase or the controls for Surgeons 1 and 3 (*p* < 0.001). However, we found significant differences between surgeons regarding the operative times of the three subgroups; the fastest manual TKAs were performed on average by Surgeon 1, while Surgeon 2 had the longest operative time (*p* < 0.001). In the learning phase, surgeries took significantly longer for Surgeon 3 than for Surgeon 1 (*p* = 0.0105), while in the proficiency phase, Surgeon 1 was faster than both Surgeons 2 and 3 (*p* < 0.01) (Figure 7).

No major complications were recorded in the RA-TKA or manual TKA groups. We recorded no wound dehiscence or infection, joint stiffness, urinary infections, central or peripheral neurologic complications, unexpected pain, or edema of the limbs. No postoperative pin insertion site infection occurred. At the time of this study, there were no documented perioperative or delayed complications related to the surgical protocol, including but not limited to acute or delayed periprosthetic infection, ligament instability, periprosthetic fracture, or thromboembolism.

## 4. Discussion

The primary objective of our study was to assess the average number of procedures needed by each surgeon to reach the inflection point between the learning and proficiency phases. Our study concluded that to achieve the proficiency state, each surgeon needed to complete a number of 6, 3, and 4 cases, respectively, for the three surgeons. We aimed to compare our findings with existing reports; however, there is a relative scarcity of literature data on ROSA-assisted TKA due to the novelty of the technology. Nevertheless, previous studies on the learning curve of other robotic systems, such as the one published by Kayani et al. [27], state that after the initial learning phase, the average RA-TKA time was similar to that of manual TKA. This finding is similar to those of several studies on RA-TKA [28,29,30,31,32]. Bolam et al. stated that the proficiency state was reached as early as after case number 5 [29]. Vanlommel et al. completed the learning curve after 6 ROSA^®^ TKAs [33], while Kayani et al. reported that 7 cases are sufficient to reach the inflection point [27]. Other reports cited longer learning curves, requiring 12 or more RA-TKA cases to transition to the proficiency phase [31,32].

Upon examination, the steep learning curve can be attributed to multiple factors, ranging from each surgeon’s experience in TKA procedures, with each surgeon performing 300+ TKA procedures annually, to the extensive resources available at our hospital dedicated solely to orthopedic procedures. The extensive experience of the surgeons can lead to confidence in the robotic-assisted procedure and the implementation of minor adjustments in the surgical technique, such as a reduced necessity for additional gap validation, quicker balancing plan of the knee, and enhanced comprehension of ROSA^®^ RA-TKA.

In our study, the average manual TKA duration was 80.56 ± 17.03 min, slightly shorter than the duration declared in other studies, such as the paper of Shah et al. [34], who reported an average surgical procedure duration of 116 ± 25 min over 1300 primary TKA interventions. In the study of Halawi et al. in 2020 [35], a number of 287 Medicare, Medicaid, and other commercial insurance TKAs were analyzed. The median intraoperative duration for TKA procedures was 113 min [35]. 

We recorded an average duration for RA-TKA of 111.54 ± 20.45 min in the learning phase and 86.43 ± 19.09 min in the proficiency phase. These durations fall within the typical time depicted in the available studies on primary TKA procedures [27,34,35]. No statistically significant difference was recorded between the operative time for the proficiency phase TKAs versus the controls represented by manual TKAs. Since no complications were recorded in these two groups, we consider that the surgeons had an acceptable manual TKA operative time, and reaching similar times in the proficiency phase was an ideal result. Literature data show that comparable operative times to manual TKA can be achieved with robotic-assisted technology within several months following its debut [28]. Moreover, longitudinal studies show that surgical times can keep improving even up to one year from the adoption of RA-TKA [36]. In their study, Weber et al. reached similar conclusions as they performed manual TKAs in an average of 77.3 min, while navigated TKAs took slightly longer, with an average of 84.1 min [37]. The similar operative times can be attributed to the fact that while RA-TKAs require some preparation, this can be performed concurrently with the surgical approach of the knee; moreover, while RA-TKA involves a series of specific steps such as pin placement, knee soft tissue evaluation, and bone reference acquisition and balancing, which are distinct from those performed during manual TKA, some surgical steps are not required, such as drilling the femoral canal or setting up an extra/intramedullary tibial guide [38,39,40]. Therefore, it is reasonable to obtain an overall similar operative time between the two methods. Nevertheless, some authors report much shorter operative times, with no significant differences regarding complications, accuracy, or patient outcomes [41].

Another particular finding of our study was the difference in operative times between orthopedic surgeons. This can be attributed, in part, to their particular medical background; type, intensity, and duration of prior medical training; and surgical mastery skills. While all surgeons benefited from the same training workshops for the ROSA^®^ system, personal surgical aptitudes can play a major role, especially when using a preferred surgical approach, as was the case in our study [42,43,44].

The constantly evolving field of medicine, especially regarding TKA surgical procedures, has raised the quality of patient care, efficiency, and precision while lowering the risk of human error and postoperative complications [22,25,45,46,47,48,49]. Incorporating robotic-assisted technologies into routine TKA procedures contributes to improvements in surgical precision, overall results, consistency, and reproducibility by offering real-time validation and step-by-step feedback. This approach ultimately leads to a personalized method, taking into consideration the patient’s knee joint anatomy and the type of prosthesis being used [38].

As expected, the introduction of a new technique to the operating theater has a learning curve and might involve complications resulting from a surgeon’s inexperience [50,51]. Frequently reported drawbacks of utilizing new modern robotic technologies in the operating room are the initial extended duration of the surgical procedure, with an inherently higher risk of surgical site infection, and the higher risk of complications in the initial learning phase [52,53,54].

Robotic systems may help improve the skills of orthopedic surgeons and the workflow of TKAs [55,56,57]. However, there are some concerns that robotic-assisted surgery may have unsatisfactory results regarding mechanical axis alignments, leading to potential deficiencies in surgical outcomes [58]. TKA accuracy relies on adequate preoperative planning and an optimal choice of implant type and approach [2,59]. Nevertheless, various studies have noted acceptable outcomes and no additional risk of complications when using ROSA^®^ compared to manual TKA [29]. Furthermore, it was shown that using this system decreases orthopedic surgeons’ stress levels and postural strain, potentially improving the ergonomics of the surgical act [60].

A challenging aspect in taking up RA-TKA is adapting to the new required steps, such as correctly acquiring the bone references, conducting the soft tissue knee state evaluation, and properly balancing the implant and knee joint. However, robotic assistance can be helpful for less experienced surgeons, and an increase in the accuracy and precision of in-training surgical fellows was observed when performing RA-TKA compared to manual TKA [61].

Another important feature of choosing RA-TKA over manual TKA is the supplementary cost associated with the robotic surgery. The cost per case was reported to be higher in RA-TKA compared to manual TKA in a recent study that included more than 4700 cases [62]. Nevertheless, a longitudinal financial analysis showed that within 3 months, costs are actually lower in robotic-assisted arthroplasty due to fewer readmissions, shorter hospital stays, and less frequent discharge into skilled nursing facilities, with patients usually going to home care [63]. The cost savings are further increased up to one year after RA-TKA, with no significant differences in complications or readmission rates [64]. These encouraging results are confirmed by the increased interest in RA-TKA, despite the high initial cost, and is preferred to manual TKA in multiple hospitals worldwide [65,66,67,68,69]. The decreased blood loss combined with superior patient outcomes and reduction in revision TKA and opioid use are strong arguments in favor of choosing RA-TKA over manual TKA [65,66,67,70].

Our study has several limitations, including the size of the study population. Only three surgeons achieved the inflection point, while the others had an insufficient number of cases needed to reach the proficiency phase. However, we included all available RA-TKA cases in order to properly assess the overall learning process and the arising complications during this period. Moreover, considering the high volume of procedures performed in our dedicated orthopedics hospital, short learning curves of the surgical staff were expected. These findings may be transferable to other tertiary centers with an intensive focus on arthroplasty but are potentially less relevant in hospitals where trauma or oncology play larger roles in the daily routine. Nevertheless, our study showed that experienced surgeons may adopt RA-TKA using this platform and quickly adapt without significant complications.

## 5. Conclusions

Robot-assisted total knee arthroplasty represents a major leap forward in orthopedic surgery. Our results suggest that this method can be quickly adopted, especially by experienced high-volume surgeons, and its implementation into routine practice may be considered achievable within a reasonable time frame. Equally importantly, we did not identify a higher rate of perioperative complications in patients subjected to robotic surgery compared to controls. In the evolving field of orthopedics, our findings support the implementation of robot-assisted technology, which can be beneficial to patients and healthcare systems alike and can improve the surgical outcomes and quality of care of patients undergoing total knee arthroplasty.

## Figures and Tables

**Table 1:** Baseline parameters in the patient group.

Parameter	RA-TKA, *n* = 39	Manual TKA, *n* = 45	*p*-Value
Age (years)	69.15 ± 5.24	67.22 ± 7.70	0.1896
BMI (kg/m^2^)	30.82 ± 4.95	30.11 ± 4.09	0.4770
Sex (male/female)	8/31	9/36	0.8306
Laterality (left/right)	18/21	23/22	0.8146
Kellgren–Lawrence classification (percentage)			
3	15	18	0.9362
4	24	27	
Limb alignment (number, HKA angle)			
Varus	39, 4.97 ± 2.27°	39, 5.00 ± 2.36°	0.9611
Valgus	-	6, 5.17 ± 2.27°	-

TKA = Total knee arthroplasty; RA-TKA = Robotic-assisted total knee arthroplasty; BMI = Body mass index; HKA = Hip–knee–ankle.

**Table 2:** Overall comparison between RA-TKA subgroups and manual TKA patients.

Parameter	Learning Phase	Proficiency Phase	Manual TKA	*p*-Value
Age (years)	68.00 ± 5.49	69.71 ± 7.89	67.94 ± 7.71	0.835
BMI (kg/m^2^)	32.35 ± 5.33	30.91 ± 7.88	30.20 ± 4.03	0.432
Sex (male/female)	4/9	2/5	6/25	0.6778
Laterality (left/right)	6/7	2/5	15/16	0.6333
Kellgren–Lawrence classification (percentage)				
3	3	3	10	0.6519
4	10	4	21	
Pre-operative varus (HKA)	5.30 ± 2.53°	5.85 ± 2.79°	4.93 ± 2.40°	0.663
Operative time (minutes)	111.54 ± 20.45	86.43 ± 19.09	80.56 ± 17.03	<0.001 ^1^

^1^ Tukey–Kramer test showed significant differences in the learning phase vs. proficiency phase and manual TKA. TKA = Total knee arthroplasty; RA-TKA = Robotic-assisted total knee arthroplasty; BMI = Body mass index; HKA = Hip–knee–ankle.

## Data Availability

The data presented in this study are available upon reasonable request from the corresponding author.

## Supplementary Materials

The following supporting information can be downloaded at https://www.mdpi.com/article/10.3390/jcm12216950/s1: Table S1: Cohen’s d effect sizes for baseline parameters comparison between RA-TKA and manual TKA in the patient group; Table S2: Cohen’s d effect sizes for pairwise comparison of parameters between RA-TKA subgroups and manual TKA patients.
