# Supplementary material for: Initial Learning Curve for Robot-Assisted Total Knee Arthroplasty in a Dedicated Orthopedics Center
Source: J Clin Med. 2023 Nov 6;12(21):6950. doi: 10.3390/jcm12216950 (PMC10649181; doi:10.3390/jcm12216950)
Supplement: Supplementary file 1 [file jcm-12-06950-s001.zip › jcm-2691886-supplementary.pdf]

**Table S1**. Cohen's d effect sizes for baseline parameters comparison between RA-TKA and manual TKA in the patient group.

| Parameter | Cohen's d effect size |
|---|---|
| Age (years) | 0.293 |
| BMI (kg/m$^2$) | 0.157 |
| Pre-operative varus (HKA) | 0.012 |

TKA = Total Knee Arthroplasty; RA-TKA = Robotic-Assisted Total Knee Arthroplasty; BMI=Body mass index; HKA=Hip-knee-ankle.

**Table S2**. Cohen's d effect sizes for pairwise comparison of parameters between RA-TKA subgroups and manual TKA patients.

| Parameter | Learning vs proficiency phase | Learning phase vs manual TKA | Proficiency phase vs manual TKA |
|---|---|---|---|
| Age (years) | 0.251 | 0.008 | 0.226 |
| BMI (kg/m$^2$) | 0.214 | 0.455 | 0.113 |
| Pre-operative varus (HKA) | 0.206 | 0.150 | 0.353 |
| Operative time (minutes) | 1.269 | 1.646 | 0.324 |

TKA = Total Knee Arthroplasty; RA-TKA = Robotic-Assisted Total Knee Arthroplasty; BMI=Body mass index; HKA=Hip-knee-ankle.
